# Spatial and temporal assessment of human-water interactions at the Inle Lake, Myanmar: a socio-hydrological DPSIR analysis

**DOI:** 10.1007/s10661-022-10730-4

**Published:** 2022-12-21

**Authors:** Kristin Peters, Paul D. Wagner, Ei Wai Phyo, Win Win Zin, Cho Cho Thin Kyi, Nicola Fohrer

**Affiliations:** 1 Department of Hydrology and Water Resources Management, Institute for Natural Resource Conservation, Kiel University, Olshausenstr. 75, 24118 Kiel, Germany; 2 Department of Civil Engineering, Yangon Technological University, Yangon, Myanmar

**Keywords:** Human-water dynamics, Socio-hydrology, Hydrologic modeling, SWAT +, Land use change, Interdisciplinary methods

## Abstract

Freshwater resources as a key aspect of socio-economic development, provide a large number of services in human and environmental systems. Nevertheless, human appropriation of these water resources and the modification of landscapes lead to potential threats on water availability and quality from local to global scales. The Inle Lake in Myanmar is an economically, traditionally, and ecologically important freshwater ecosystem that faced severe degradation from the 2000s. In its catchment area, a Driver-Pressure-State-Impact-Response (DPSIR) framework is applied for an assessment period of 30 years from 1990 to 2020. The analysis results are complemented with a socio-hydrological survey, water quality assessment, a land use classification based on ground truth and satellite data, and hydrologic models. The resulting land use changes, − 13% forest, + 13% agriculture, and + 5% urban areas, lead to increased water yield, decreased evapotranspiration, and increased sediment yield. Together with other drivers and pressures such as climate change and anthropogenic pollution, these human activities are major threats for freshwater resources and the ecosystem. However, the existing awareness of the local population for the environmental degradation is obstructed by national and international crises and responses to negative developments can accelerate degradation if they are unplanned and short-term solutions. Our study shows that environmental degradation processes have a complex nature and can only be tackled in a coordinated way with a long-term perspective. DPSIR is a suitable approach to assess human-water dynamics and disentangle the complex interconnectedness of social and environmental systems in freshwater ecosystems, even in data-scarce regions.

## Introduction

Water resources are naturally recharged through the water cycle, but the availability of freshwater for human and ecosystem needs is threatened (Oki & Kanae, 2006; Reid et al., 2019). Worldwide, the freshwater resources, such as rivers and lakes, account for about 0.01% of global water (Dudgeon et al., 2006). The available sources are threatened by biodiversity loss, pollution, and increased withdrawals, affecting nearly 80% of the world’s population (Dudgeon et al., 2006; UNESCO, 2021; Vörösmarty et al., 2010). Freshwater lake ecosystems are among the most vulnerable and heavily used systems often with a historical legacy of degradation (Beklioğlu et al., 2016; Zhang et al., 2018). This degradation is mainly caused by anthropogenic activities, such as land use changes, increasing population, urbanization, overexploitation, other transformations of natural water systems, and is further accelerated by climate change (Foley et al., 2005; Reid et al., 2019). It is projected that by 2050 water scarcity is one of the main global problems with a strong local relevance especially in the Global South (Boretti & Rosa, 2019). Furthermore, the ineffectiveness to target individual drivers increases the need to understand the—often nonlinear—relationships in complex socio-ecological systems for developing sustainable management strategies (Ormerod et al., 2010; Smith et al., 2015). The complexity of these cause-effect relationships requires an interdisciplinary research approach of combining social and natural sciences (Boretti & Rosa, 2019; Di Baldassarre et al., 2019; Re et al., 2021; Rockström et al., 2009).

To assess the causal interconnections between the possible influences on water resources, the Driver-Pressure-State-Impact-Response (DPSIR) framework (Kristensen, 2004) is applied. This “causal framework for describing the interactions between society and the environment” (EEA, 2020) was applied worldwide for the assessment of human activities and their effects on ecosystems (Feld et al., 2010; Gari et al., 2018; Gebremedhin et al., 2018; Newton & Weichselgartner, 2013; Patrício et al., 2016; Snoussi et al., 2007; Sun et al., 2016; Wang et al., 2015). It was implemented in several countries as a tool for providing a decision basis for policy makers in the field of water resources management (Gebremedhin et al., 2018; Mehryar et al., 2017). Sun et al. (2016) applied the framework for the city of Bayannur in China, finding decreasing water resources system sustainability caused by high water consumption and pollution. The application of DPSIR in freshwater lake ecosystems (Gebremedhin et al., 2018; Janssens de Bisthoven et al., 2020) has shown how anthropogenic influences such as overfishing, land use changes, or mining can cause degradation of the ecosystem.

The study area of the Inle Lake catchment is located in central Myanmar and represents a sensitive freshwater lake ecosystem with connections to various human influences on a local, national, and global scale. Myanmar has been cited as the second most affected country by climate change over the past two decades (Eckstein et al., 2021) and extreme events such as floods and droughts are predicted to mainly influence the central dry zone (Rao et al., 2013). Since the country faced political, economic, and demographic changes after the end of political isolation in 2011, it is assumed that subsequent socio-economic changes have large impacts on water resources (Kattelus et al., 2014; Taft & Evers, 2016). The 30-year assessment period from 1990 to 2020 can be divided into a period before and after the political liberalization in 2011 that came to a halt in 2021. From the 2000s, accelerating degradation of the water quality and reduction of the Inle Lake’s depth and open water area have been observed by the local community and documented in scientific reports (Furuichi, 2009; Khaung et al., 2021; May, 2007; Phyo et al., 2019; Pradhan et al., 2015; Sidle et al., 2007; Thin et al., 2016). The lake is of particular importance for its inhabitants since they strongly depend on the ecosystem services it provides, such as the provision of clean drinking water from groundwater sources. Re et al. (2021) applied a socio-hydrogeological approach to understand the water user’s perspective while undertaking a hydrogeochemical assessment. The combination of household interviews and scientific field measurements highlights the need for integrating social and environmental methods at this study area and for extending this approach to a broader perspective.

In our study, we particularly aim to identify local, national, and global human influences on sensitive freshwater ecosystems and the relationships among them at catchment scale. For this purpose, the study area of the Inle Lake catchment serves as an exemplary study area for a sensitive freshwater lake ecosystem in a data-scarce region. The historical traditions and high complexity in this human-environmental system make it necessary to assess it in a structured framework. The combination of DPSIR with a hydrologic model and the results from intensive field and literature studies can quantify relevant indicators at the study area and provide new results for local stakeholders. Furthermore, our study shows that the applied combination of interdisciplinary methods can be applied to comparable data-scarce freshwater ecosystems under pressure of human activities.

## Materials and methods

### Study area

**Fig. 1:**
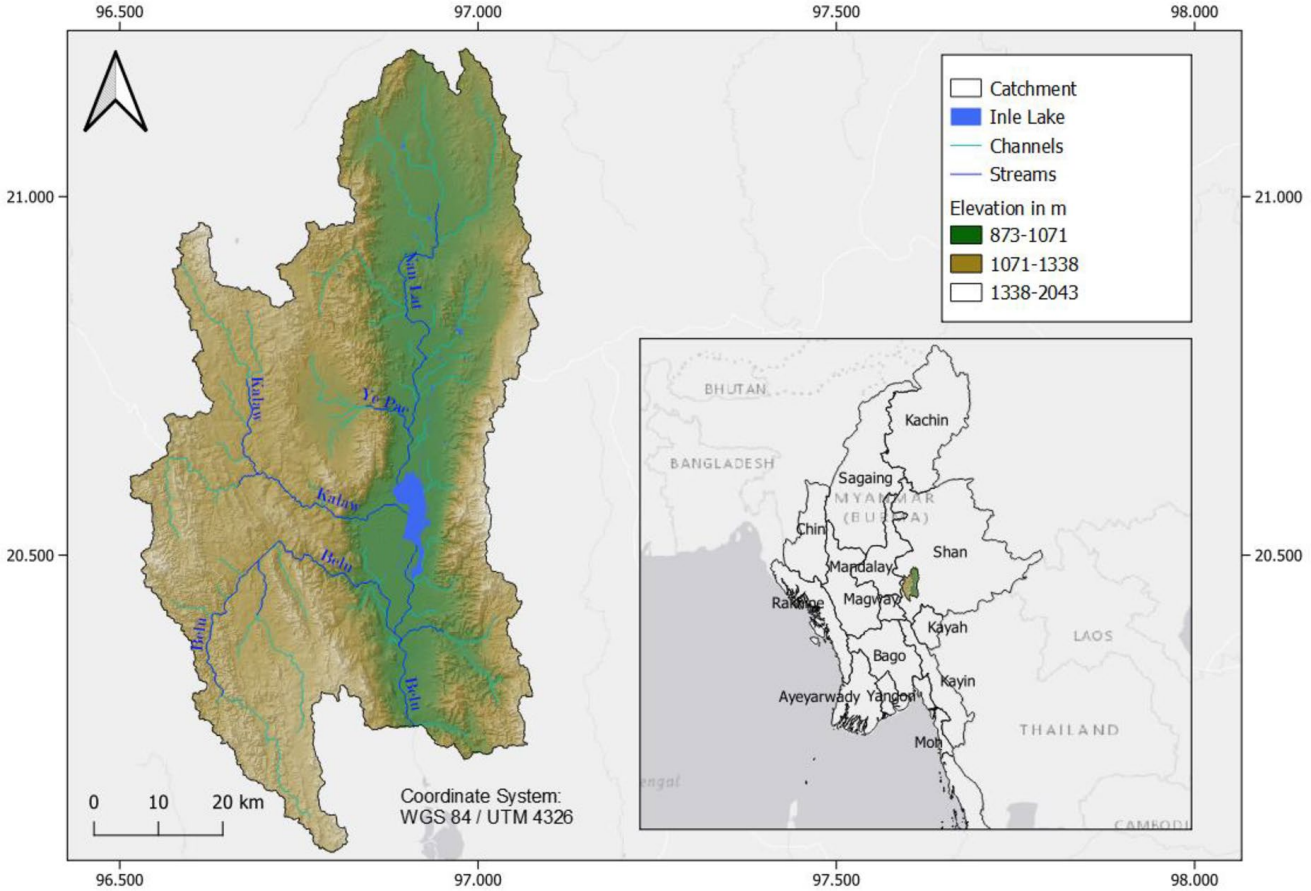
Location, elevation (Jarvis et al., 2008) with hillshade effect and main river system of the Inle Lake catchment

Inle Lake, also known as Inlay Lake, is the second largest natural lake of Myanmar, situated 884 m above mean sea level on the Shan plateau, flanked by two mountain ranges to the east and west (Ballot et al., 2017) (Fig. 1). Inle Lake’s dimensions are discussed in several reports (Ballot et al., 2017; Michalon et al., 2019; Sidle et al., 2007), due to seasonal variations and definition issues regarding the inclusion of floating gardens on the lake. In this study, the lake area refers to the open water area that is not covered by hydroponics or wetland. The total area of the shallow freshwater lake was defined within the scope of this study by a land use classification of 2020 resulting in a value of 38 km^2^, whereas the whole lake area in 2020 including floating gardens, stilt houses, and wetlands accounts for about 128 km^2^.

Geology of the Inle Lake catchment is characterized by Lower Paleozoic to Tertiary carbonate and clastic sedimentary rock covered by Terraosa soil and Tertiary lacustrine sediments (Aung et al., 2019). Mineralogical characteristics of the sediments are constituted by the Thitsipin Limestone Formation and Nwabandgy Dolomite Formation (Sacchi et al., 2017). Typical soil profiles on the lower slopes consist of clay on top of a 40–45cm clay and loam texture with underlying bedrock of limestone and calcareous sandstone and siltstone, described as Acrisols and red-brown soils (Sidle et al., 2007). The Inle Lake catchment experiences a tropical savannah (Aw) climate in the south and is dominated by a temperate climate (Cwa) beginning with the mountainous areas in northern and eastern Myanmar according to the Köppen-Geiger climate classification (MIMU, 2018). The region has three prevailing seasons: a cold dry winter from November to February, a hot dry summer from March to May, and a rainy season from June to October. The average annual temperature is between 23 and 35 °C (Ballot et al., 2017). Data from the Department of Hydrology and Meteorology (DMH) shows that precipitation is generally higher in mountainous regions in the south of the catchment (~ 2200 mm/a at Pinlaung weather station) and lowest in the flat regions around Heho (~ 1100 mm/a at Heho weather station) (DMH, 2019). About 70% of the annual rainfall is received in the months from July to September (Sidle et al., 2007).

Since 2015, Inle Lake belongs to the UNESCO World Biosphere Reserves, due to its cultural and biological relevance. It is estimated to have formed 1.5 million (ICIMOD & MONREC, 2017; Karki et al., 2018) to 65 million (Aung et al., 2019) years ago and the lake and its wetlands inhabit several endemic species (Annandale, 1918a, b). The lake is a habitat for a unique biodiversity and at least 31 different species were identified in 1918 (Annandale, 1918a): among several native Asian species, also endemic ones, of which two (*Systomus compressiformis* and *Silurus burmanensis*) are assumed to be already extinct (Kano et al., 2016). Humans are estimated to have inhabited the Inle Lake region since the Neolithic Age (UN Habitat, 2014). Nowadays, the inhabitants’ livelihoods include commercial fishing, textile industry, smithy, land-based and floating agriculture, aquaculture, and tourism (UN Habitat, 2014). Industrial sectors in the catchment were found to be coal mining, cement, and textile production. Declared as an ASEAN heritage site in 2003 (Khurtsia, 2015), the Inle Lake became an increasingly popular tourist destination. Inle Lake is famous for its practice of hydroponics, also called *Yechan*. A practice that is widely used for its high productivity in natural and artificial environments (Jensen, 1997). The floating fields consist of water hyacinths (Michalon, 2014), soil from the wetlands, and seaweed (Sidle et al., 2007). The major cash crop grown in the floating gardens is tomato, often in several seasons. Lowland cultivation is mainly made up of rice fields in the vicinity of the lake (Htwe, 2015). Within this region, rice is cultivated often in rainy and dry season, whereas in the dry season, the lake water is pumped to the fields for irrigation (Personal Interviews, 2020) . In the mountainous regions to the west and east of the lake, shifting cultivation or *Taunggya* is nowadays still practiced, which involves clearing the forest with controlled fires—slash and burn (Htwe, 2015)—prior to the rainy season with the purpose to release nutrients for a period of years (Sidle et al., 2007).

### Lake hydrology and water quality

In total, four major streams (Fig. 1) drain into the lake and its surrounding wetlands: Nan Lat from the north, Ye Pae from the north-west, Kalaw from the west, and Belu from the south-west. The outflow connects to the downstream Saga lake, serves the hydroelectric power plant of Lawpita (May, 2007), and further downstream the Thanlwin river (Khaung et al., 2021). The volume of Inle Lake is defined by Ballot et al. (2017) with a maximum depth of 3.7 m and a mean depth of 1.52 m. The average annual inflow is estimated to be 1.1 × 10^8^ m^3^ per year, maximum water storage capacity of 3.5 × 10^7^ m^3^, no stratification (Thin et al., 2016), and a residence of 0.32 years (Khaung et al., 2021). Although high concentrations of nutrients could be assumed from floating garden agriculture and the intensifying agriculture in the surroundings (Akaishi et al., 2006), findings from recent water quality assessments show medium concentrations of nitrogen and phosphorous (Ballot et al., 2017; Re et al., 2021). The high resilience to accumulation of anthropogenic inputs of the lake is caused by its low residence time and calcite precipitation causing the removal of phosphorous (Re, et al., 2018; Thin et al., 2016). The lake is calcareous with an average calcium value of 49 mg Ca/l (Ballot, et al., 2017). Nevertheless, Swe and Hnin (2013) classified the lake as eutrophic based on nitrogen concentrations of above 600 µg/l in dry season of 2009 and 2010 and mesotrophic to semi-eutrophic by Ballot et al. (2017) with a mean of about 480 µg/l and higher nutrient values in the streams. The nutrients are assumed to be mainly taken up by aquatic macrophytes on the lake bottom and therefore not available for phytoplankton growth (Ballot et al., 2017). Total phosphorous (TP) was measured with mostly low values and is assumed to be stored in bottom sediments, acting as a sink (Thin et al., 2020a). Two distinct P sources were identified: non-bioavailable calcium-bound P (Ca-P) from anthropogenic sources and organic-P (OP) from detrital input related to soil erosion, which is more labile. Organic matter degradation creates a risk for this P release and a shift to anoxic conditions due to increasing climate changes and anthropogenic activities. Contamination with heavy metals is another threat to water quality of Inle Lake. The assessment of heavy metals in sediments resulted in high values for arsenic (As) and antimony (Sb) (Aung et al., 2019) presumably from anthropogenic sources. Groundwater contains high concentrations of iron (Fe), manganese (Mn), and As (Re et al., 2021). Mn values were found as well by Thin et al. (2020b), but with main sources from natural weathering processes and lithogenic origin. The most abundant metal in sediments according to Thin et al. (2020b) was calcium (Ca).

### DPSIR approach

**Fig. 2:**
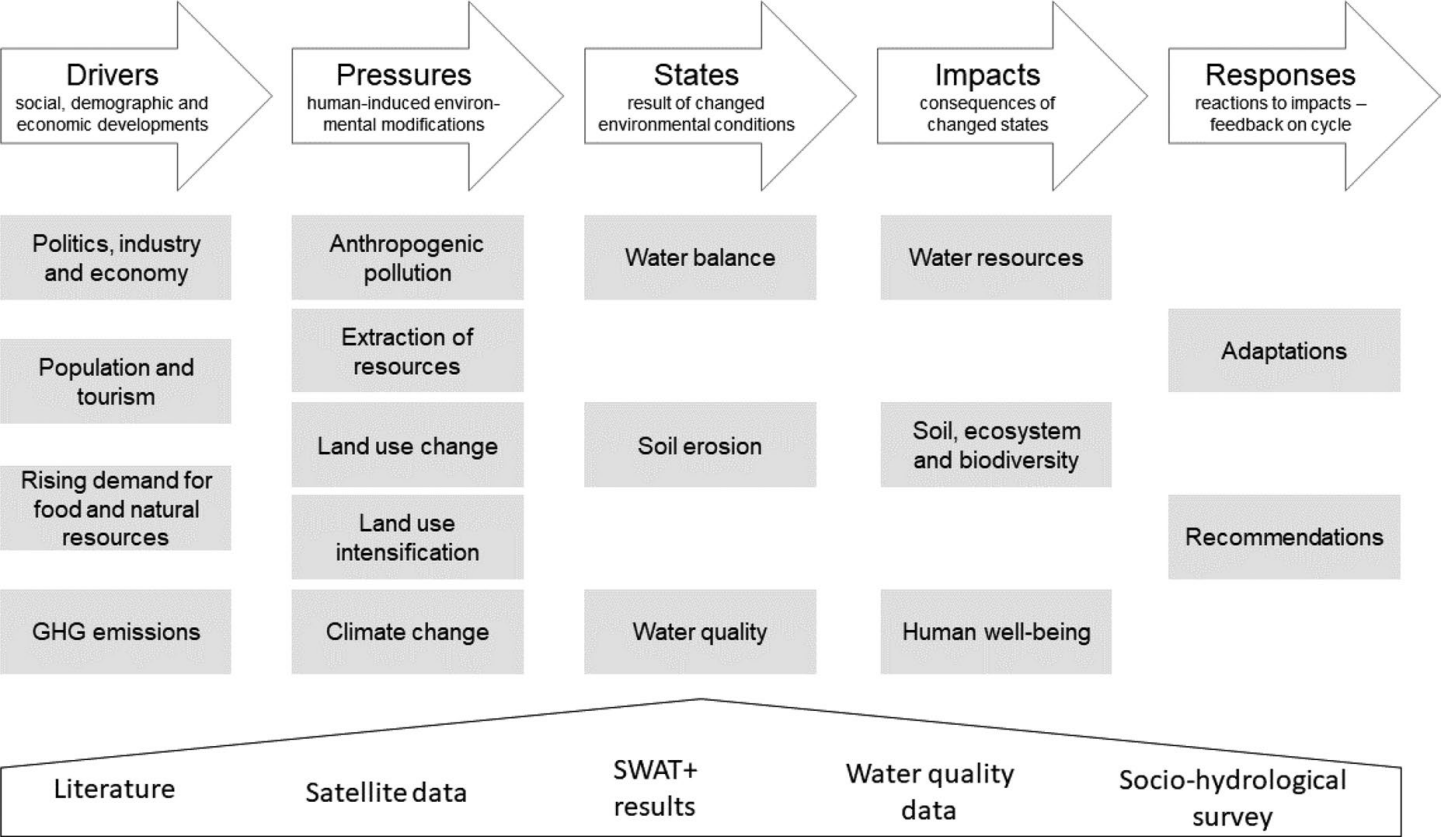
The DPSIR framework applied to the Inle Lake

Inle Lake is influenced by many different physical and socio-economic factors. To assess the causal connections between the sources of stressors and their effects, it is suitable to apply a multidisciplinary framework. DPSIR is a “causal framework for describing the interactions between society and the environment adopted by the European Environment Agency: driving forces, pressures, states, impacts, responses (extension of the PSR model developed by OECD)” (EEA, 2020). It was developed by Kristensen (2004) for the EEA as a structuring instrument for reporting and to consult policy makers on environmental impacts (Kristensen, 2004). The framework was widely applied for different environmental topics to assess water quality (Gari et al., 2018) and water resources or coastal vulnerability (Newton & Weichselgartner, 2013). It was applied as an eDPSIR (enhanced DPSIR) framework by Taft and Evers (2016) for the whole country of Myanmar with focus on human-water dynamics. These various applications lead to different viewpoints and approaches, which make it possible or necessary to amend the framework. This has been done by Müller and Burkhard (2012), who include impacts on ecosystem services in an adaptive management cycle (Müller & Burkhard, 2012) or by Elliott et al. (2017) who optimized the framework by including activities, human welfare, and measures in the DAPSI(W)R(M) (Elliott et al., 2017). Nevertheless, DPSIR is criticized to oversimplify complex environmental problems and terms are often not consistent in different studies (Patrício et al., 2016). For this study, the basic framework described by Burkhard and Müller (2008) is assumed to be applicable for analyzing the interactions within the human-environmental system with special regard on water resources of Inle Lake and its catchment. In the framework, it is assumed that social, demographic, and economic developments take place (Müller & Burkhard, 2012) and human demand exists for goods and products (Burkhard & Müller, 2008). This defines the drivers, such as socio-economic development, leading to certain pressures. The pressures can be described as human-induced modifications of the environment, such as the release of polluting substances or the utilization of land and resources (Müller & Burkhard, 2012). One of the pressures, climate change, has a special position, due to its complex and global character. It is therefore often regarded as an external factor influencing the human-environmental system (Burkhard & Müller, 2008). Resulting from the human activities, described by the pressures, the states are the result of changed environmental conditions. To assess the state of the environment in a holistic manner, Burkhard and Müller (2008) recommend the application of modeling tools. Changes in these environmental states lead to impacts on water resources, the environment, and human well-being. Indirect relationships and delayed reaction time between the pressures, states, and impacts are possible and make it difficult to define them (Burkhard & Müller, 2008). Nevertheless, this part is important, because it shows the consequences of human actions on the environment, which lead to certain responses. Within the DPSIR framework, the responses lead back to the drivers and close the cycle. They also lead to changes in pressures and states, which makes it possible to observe the effectiveness of management actions. Responses are referred to as “process indicators” (Burkhard & Müller, 2008). With the help of this basic DPSIR framework, we aim to provide baseline information for a data-scarce region and identify the most significant stressors in the human-water dynamics based on data from literature, socio-hydrological field assessment, satellite and water quality data, and hydrological modeling (Fig. 2).

### Socio-hydrological field assessment

**Fig. 3:**
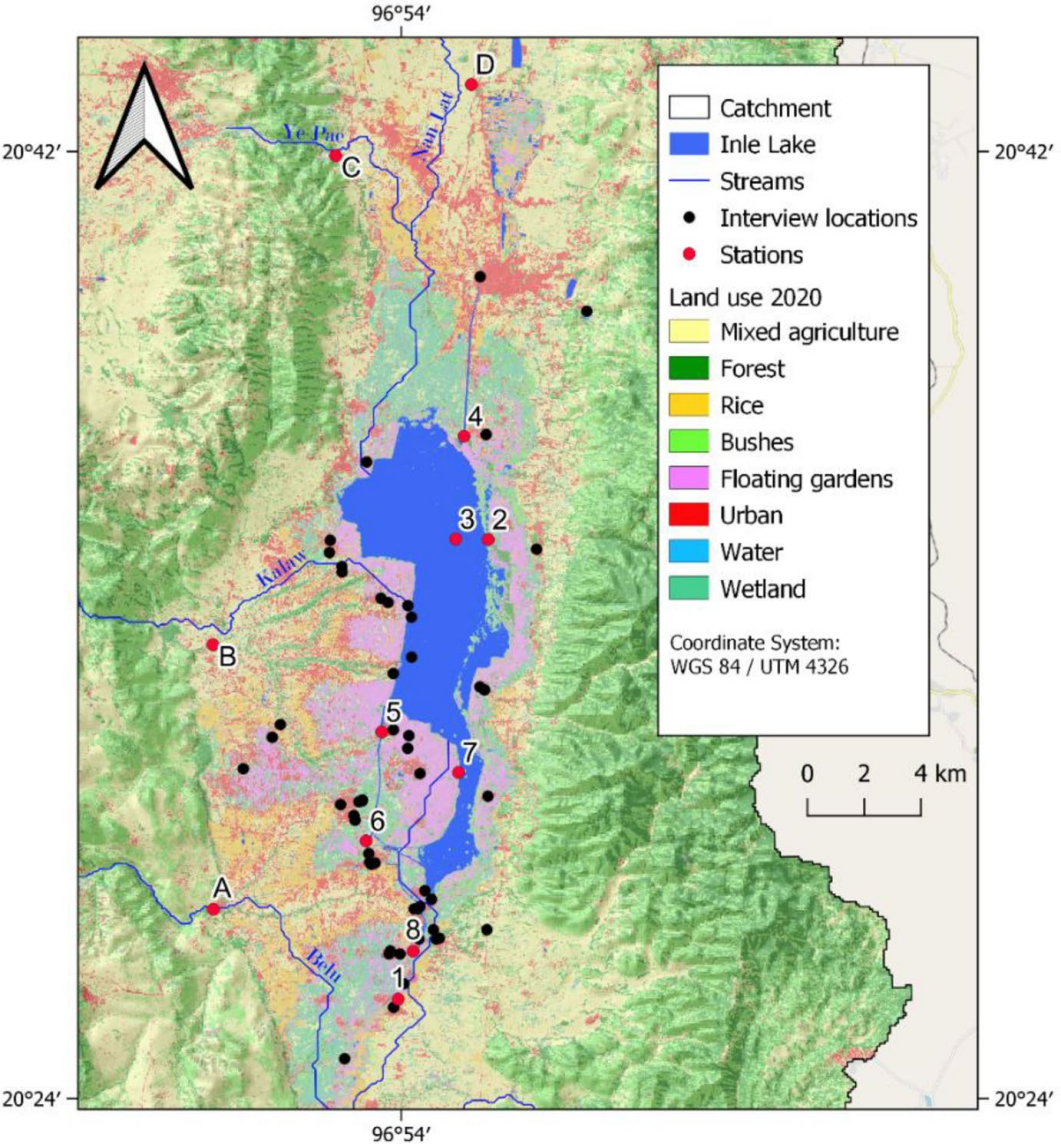
Locations of sample points and interviews in the study area with land use, hillshade effect, and streams delineated from SWAT +

A quantitative socio-hydrological survey among 148 residents of the lake area with different professions (Table 1) has been conducted to assess the above-mentioned changes of the environment and people’s behavior towards it. Additionally, 15 tourists were questioned in a qualitative way. The interviews took place from 8 to 14th of March 2020 in *Nyaung Shwe* and in the villages on the lake and its surrounding. The semi-structured quantitative survey with 35 questions concerning people’s awareness of the environmental changes, effects of degradation, and daily practices was prepared in English and translated to Myanmar language. The local people were semi-randomly selected (Young et al., 2018), by asking people who were traveling along by boat or encountered on markets or other public locations (Fig. 3). Even though a randomized selection approach is not the most common one used in conservation science (Young et al., 2018), it was chosen due to its practicability in the study area. It has especially been frequently used for assessing human impacts on the environment (Ingram et al., 2011; Kipkemboi et al., 2007). The locations were preselected to represent the geographical distribution of the main villages and to achieve a representative sample of professions. The goal of the survey is to include the findings in the DPSIR framework and to make assumptions for respective drivers, pressures, states, impacts, and responses and possible connections between them (Annex). All participants were informed about the scientific purpose of the study and participated voluntarily.

**Table 1:** Socio-economic profile of local survey participants

**Category**	**Sub-category**	**Frequency**	**Percentage**
**Age**	15–30	44	29.7%
	30–50	60	40.5%
	50–70	40	27.0%
	> 70	4	2.7%
**Gender**	Male	50	33.8%
	Female	81	54.7%
	N.A	17	11.5%
**Profession**	Service	22	14.9%
	Seller	33	22.3%
	Farmer	48	32.4%
	Weaver	9	6.1%
	Smith	4	2.7%
	Fisher	4	2.7%
	Artisan	9	6.1%
	Other	19	12.8%
**Village**	Lake, N	3	2.0%
	Lake, W	37	25.0%
	Lake, E	18	12.2%
	Lake, S	50	33.8%
	Outside of lake	40	27.0%
**Total**		148	100.0%

**Fig. 4:**
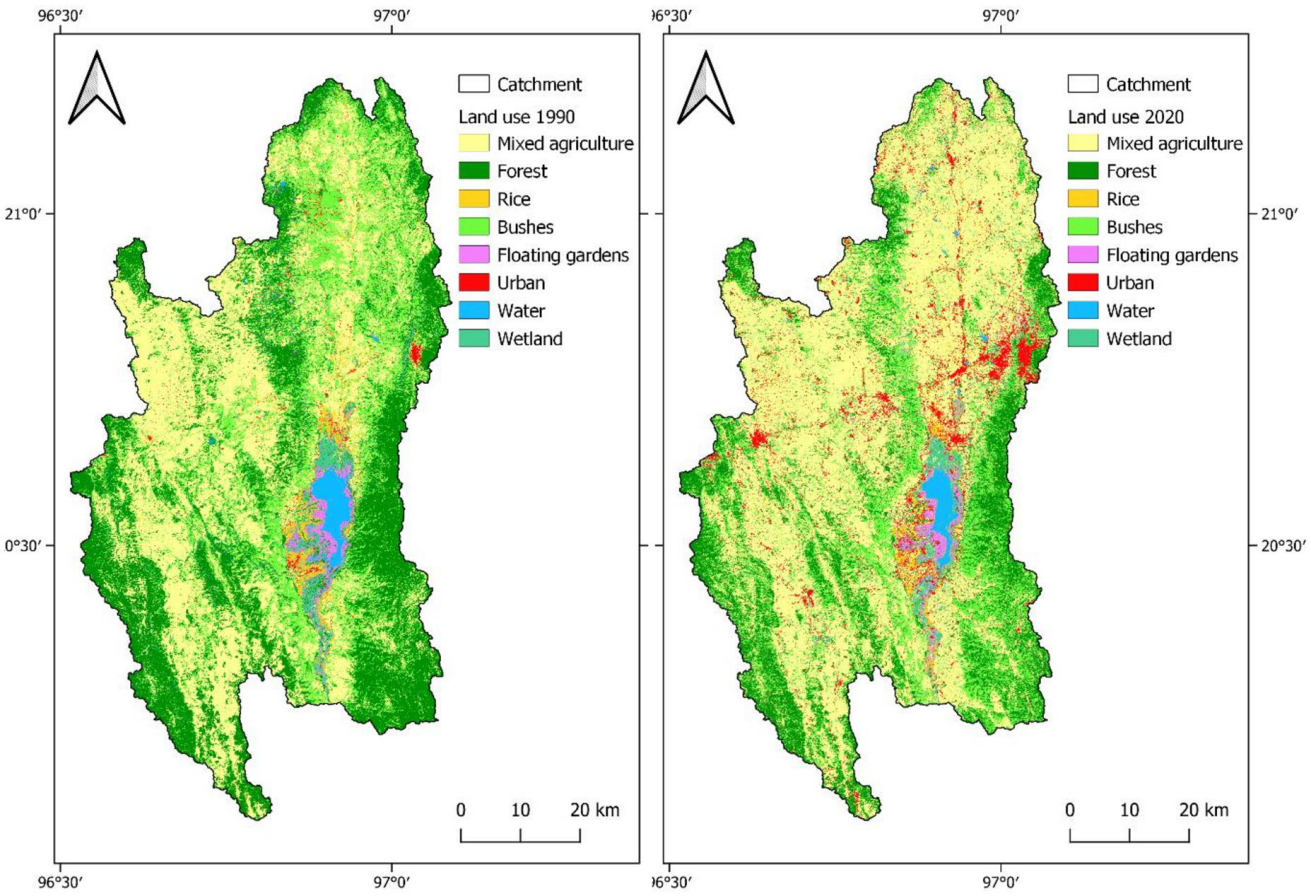
Land use maps of 1990 and 2020

### Water quality assessment

For the water and sediment sample collection and the measurement of in situ parameters, 12 stations were selected (Fig. 3). In situ data was measured for pH, conductivity, and total dissolved solids (TDS) with multi-parameter probes Lovibond and Multi 340i from a boat in about 20-cm water depth in a time period between 9 am and 1 pm on 15th and 16th of March. The four locations at the streams were measured from the shores or from a water sampler. Sediment samples were collected on 9th March at the 8 stations on the lake. The sediment was dried for 4 days, under the given circumstances from 7 am to 7 pm at a mean air temperature of 30 to 35 °C. Afterwards, the sediment was sieved to about 2-mm particle size in a plastic sieve to remove large organic parts such as seaweed or shells. The samples were stored in separate plastic bags for each station for about 4 months before they were grinded with a planetary mill and analyzed with an EuroEA Elemental Analyzer for C%.

### *Hydrological modeling with SWAT* + 

The Soil and Water Assessment Tool (SWAT) is a catchment scale model to assess the influence of changing land use on water, sediment, and agricultural yields (Arnold et al., 1998). The hydrological model is a tool to simulate current and future hydrological processes in the study catchment and to assess land use (Fohrer et al., 2005; Tigabu et al., 2019; Wagner et al., 2013) or climate change effects (Mahmoodi et al., 2020; Tigabu et al., 2020). It has been applied for many different regions with limited data availability (Wagner et al., 2011) such as India (Wagner et al., 2013), the Himalayan and tropical basins of Asia (Shrestha et al., 2018), Myanmar (McGinn et al., 2021; Shrestha et al., 2014, 2017), or China (Bieger et al., 2014). Furthermore, it was used to model sediment yield for regions with no measured long-term sedimentation data with good performance results (Pacetti et al., 2020; Schmalz et al., 2015). It is therefore a suitable model to assess the characteristics in the Inle Lake catchment for the determination of pressures and states in a DPSIR framework. The model is applied with the goal of assessing the effects of a changed land use from 1990 to 2020 on the water balance components, evapotranspiration (ET) and water yield (WY), and on sediment yield (SY) in the catchment. The SWAT + model (version 60.5) has been used, and the QSWAT + Plugin for QGIS and the SWAT + Editor have been used for the model setup. Based on the DEM, obtained from the Shuttle Radar Topographic Mission (CIAT-CSI SRTM 30 m resolution) (Jarvis et al., 2008), the catchment was divided into 15 subbasins by setting the stream threshold to 200 km^2^. The outlet of the catchment was set manually at the narrowest point at the Inle Lake outlet since there is no gauging station for observed discharge data. The watershed delineation creates a total catchment size of 4368.25 km^2^. Moreover, based on the terciles of the slope class distribution, three slope classes were defined as < 2.8%, ≥ 2.8%, and ≥ 8.3%. We used SoilGrid data (soilgrids.org), which is based on soil profile observations from the World Soil Information Service (WoSIS) database and mapped using machine-learning algorithms. The soil map including physical and chemical soil characteristics for SWAT + was derived using the R-Script with soilgridr (Schuerz, 2019).

Land use maps were produced for the years 1990 and 2020 based on Landsat 5 (30-m spatial resolution) and Sentinel 2A (10 m) sensor images (Fig. 4). Eight land use and land cover (LULC) classes are included: agricultural land generic (AGRL), deciduous forest (FRSD), rice (RICE), range-brush (RNGB), floating gardens/tomatoes (TOMA), urban (URBN), water (WATR), and wetland (WETL). During the field assessment in March 2020, reference polygons were mapped as ground truth for a supervised classification using the random forest algorithm (Breimann, 2001; Liaw & Wiener, 2002). To improve the classification accuracy, polygons were added for known land use classes, transferred to the 1990 satellite image, and verified using false-color imagery. Ground truth data for both years was randomly split into training and test dataset. The overall accuracy is 75.77% in 1990 and 76.38% in 2020. In addition, the reliability or user’s accuracy shows the probability that the sample indeed represents the assigned land use class is given in Table 2 (Story & Congalton, 1986).

**Table 2:** User’s and overall accuracy of the land use classifications

	User’s accuracy	
LULC class	1990	2020
Mixed agriculture	88.01%	83.95%
Forest	80.38%	76.80%
Rice	60.14%	51.87%
Range-brush	59.66%	68.06%
Floating gardens/tomatoes	76.42%	85.83%
Urban	54.37%	76.00%
Water	90.16%	88.54%
Wetland	65.22%	71.67%
Overall accuracy	75.77%	76.38%

Plant parameters for FRSD were adjusted to better represent plant growth in the study area. In particular, the potential leaf area index (LAI) (lai_pot) and the minimum LAI (lai_min) were increased to better represent the tropical characteristics with higher forest canopy cover based on MODIS satellite images for LAI. To overcome the model’s assumption of dormancy related to the shortest day in the year, which is not applicable in the tropics (Wagner et al., 2011), management schedules were used to avoid the dormancy period and better fit the conditions in the catchment (Strauch & Volk, 2013). For the three agricultural classes AGRL, RICE, and TOMA, a management schedule was created to represent the land use practices more accurately. RICE and TOMA include a crop rotation: On a rice field, corn is planted during the dry season and on the floating tomato gardens, a second crop is eggplant (Personal Interviews, 2020).

Measured weather data is available for three stations within the catchment: *Pinlaung* in the south of the catchment, *Taunggyi* in the east, and *Heho* north-west to the Inle Lake. Precipitation data could be obtained from the Department of Meteorology and Hydrology (DMH) with daily values from 1995 to 2019, as well as temperature data with minimum and maximum values for the same period. Since no other measured data was available, the Hargreaves method was selected to calculate potential evapotranspiration in SWAT + , because it requires only temperature as an input and it has been applied successfully in other SWAT studies in Myanmar (McGinn et al., 2021; Sirisena et al., 2018).

After adding the input data, the number of hydrological response units (HRUs) was 11,915 in 2020 and 11,076 in 1990 with no exclusion of any land use, soil, or slope class. To assess the effect of land use change between 1990 and 2020 on water balance components, two exactly similar models were created with the settings described below, except for the two different land use maps. The water balance equation is the basis for the hydrologic cycle simulated by the SWAT model (Neitsch et al., 2011). The analysis of its components evapotranspiration and water yield in the two different models on catchment, subbasin, and HRU level allows assumptions on the influence of land use changes on water availability. Both models were run with the available weather data for a spin-up phase of three years from 01/01/1995 to 31/12/1997, and a 22-year observation period until 28/12/2019 at a daily time-step.

#### Sediment calibration

The sediment yield in SWAT + is calculated with the Modified Universal Soil Loss Equation (MUSLE (Williams, 1995)) and refers to the field erosion.

**Equation ****1** Modified Universal Soil Loss Equation (MUSLE (Neitsch et al., 2011))

1 $$\begin{aligned}sed=&11.8*{\left({Q}_{\mathrm{surf}}*{q}_{\mathrm{peak}}*{\mathrm{area}}_{\mathrm{hru}}\right)}^{0.56}*{K}_{\mathrm{USLE}}\\&*{C}_{\mathrm{USLE}}*{P}_{\mathrm{USLE}}*{LS}_{\mathrm{USLE}}*CFRG\end{aligned}$$

The simulated process includes the surface runoff volume (${Q}_{\mathrm{surf}})$, peak runoff rate (${q}_{\mathrm{peak}})$, the area of the HRU (${\mathrm{area}}_{\mathrm{hru}})$, the USLE factors soil erodibility (${K}_{\mathrm{USLE}}$), cover and management (${C}_{\mathrm{USLE}}$), support practice (${P}_{\mathrm{USLE}}$), topographic factor (${LS}_{\mathrm{USLE}}$), and the coarse fragment factor ($\mathrm{CFRG}$) (Neitsch et al., 2011). The values used in the SWAT + model 2020 for the USLE parameters are listed in Table 3. ${K}_{\mathrm{USLE}}$ and ${C}_{\mathrm{USLE}}$ have been adopted from Htwe et al. (2015), who derived the parameter values from field observations and calculations in the Inle Lake catchment.

**Table 3:** USLE parameter settings in SWAT +

**Factor**	**SWAT + default**	**Calibrated value**	**Source**
*K*_*USLE*_	0.65	0.02	(Htwe et al., 2015)
*C*_*USLE*_	AGRL: 0.2	0.45	(Htwe et al., 2015)
	TOMA/EGGP: 0.03	0.01	(Htwe et al., 2015)
	RICE/CORN: 0.03/0.14	0.35	(Htwe et al., 2015)
	RNGB: 0.003	0.01	(Htwe et al., 2015)
	FRSD: 0.001	0.001	(Htwe et al., 2015)
	WETL: 0.003	0.01	(Htwe et al., 2015)
lat_sed	0–5000	18.0	Calibrated (in comparison to 41.4–62 (Aghsaei et al., 2020))

After setting these values according to Htwe et al. (2015), the SWAT + model result for average annual sediment yield was with 4 t/ha well below the value of 16.7 t/ha in 2009 estimated by Htwe et al. (2015) and 14.2 to 54.6 t/ha by Tun et al. (2015). An estimated value of 1.5 to 1.8 t/ha was assumed by Sidle et al. (2007) to be mainly applicable for undisturbed forests but not for the agro-ecosystems in the Inle Lake catchment, where the contemporary sediment yield is presumably higher. The sediment yield was therefore calibrated for the higher result from Htwe et al. (2015) using the parameter lat_sed, which was used successfully in many SWAT studies (Aghsaei et al., 2020; dos Santos et al., 2020). It describes the sediment concentration in lateral flow and groundwater flow in mg/l and was set by Aghsaei at el. (2020) to 41.4–62 mg/l. After manual calibration with 25 test runs, a value of 18.0 mg/l provided a final result of 16.7 t/ha average annual sediment yield which was the closest to the literature value. After calibration of the 2020 model, the same settings were adopted for the 1990 model. The results for sediment yield of the SWAT + model presented in this work can therefore provide reliable estimations since the model has been proven to perform well in sediment calculation even in data-scarce regions (Pacetti et al., 2020; Schmalz et al., 2015).

#### Model evaluation

Application of hydrological modelling tools generally requires a high density of measured environmental data to validate predictions. Nevertheless, technological advances have increased the process understanding of the hydrological community and amplified initiatives to work with ungauged basins (Sivapalan et al., 2003). The process-based model SWAT has been proven to work well in data-scarce regions (Bieger et al., 2014; Schmalz et al., 2015) and ungauged basins (Athira et al., 2016) and since there is no measured discharge data from the outflow of Inle Lake to validate the SWAT + model, the 2020 model’s water balance components precipitation, water yield, and evapotranspiration were evaluated.

The runoff coefficient is a factor that is commonly used to convert precipitation to runoff and depends on the properties of soil and vegetation. It has been set for typical land uses by the American Society of Civil Engineers and Water Pollution Control Federation. For forest, it has a value of 0.05 to 0.2 and for urban areas a value of 0.3 to 0.5 (Goel, 2011). Since the properties of soil and land use highly vary among different catchments, it was decided to compare three catchments in the proximity to the Inle Lake catchment: *Chindwin*, *Toungoo*, and *Bago*. The runoff data was retrieved for all catchments from GRDC (Global Runoff Data Centre). The precipitation data for *Bago* and *Toungoo* catchment was produced from the National Centers for Environmental Prediction (NCEP) Climate Forecast Reanalysis (CFSR) mission. The precipitation data for *Chindwin* was taken from McGinn et al. (2021), which is based on CFSR data (McGinn et al., 2021). In Table 4, it is observable that precipitation differs strongly among the catchments, which is caused by the different climate zones and elevations in Myanmar.

**Table 4:** Comparison of runoff coefficients in Myanmar catchments

	**Inle Lake**	**Toungoo**	**Chindwin**	**Bago**
**Catchment size (km**^**2**^**)**	4368	14,543	115,300	3170
**Precipitation (mm/a)**	1514.01	2210.75	2848.40	3093.01
**Water yield (mm/a)**	413.42	661.24	1280.10	1617.93
**Runoff coefficient**	0.27	0.30	0.45	0.52

*Toungoo* is most comparable to Inle Lake being the closest catchment with a coefficient of 0.3. Also comparable in size but located further in the south close to *Irrawaddy* delta and in a tropical monsoon climate is the *Bago* catchment with a high value of 0.52. The value of 0.45 in the *Chindwin* catchment also covers a large area with diverse tropical land use and climate (McGinn et al., 2021). Considering these overall differences from the Inle Lake catchment, the ratio of 0.27 is assumed to be plausible. In addition, the modeled average annual evapotranspiration was compared to the actual evapotranspiration from the Global Land Evaporation Amsterdam Model (GLEAM version 3.5a, 2020; Martens et al., 2017; Miralles et al., 2011) For the Inle Lake catchment, the data has a significant correlation (*p* = 0.001, *r*^2^ = 0.4) for the observation period from 1998 to 2019.

## Results and discussion

### Drivers

#### Politics, industry, and economy

The 30-year study period from 1990 to 2020 can be divided into a period before and after the political liberalization in 2011. After a long period of political and economic isolation, the political liberalization in 2011 led to an ongoing democratization process, which came to a halt in 2021. Since 2011, the investments from neighboring countries, aid organizations, and donor funds had increased rapidly (Bjarnegård, 2020; Taft & Evers, 2016). Before, western donors refused to support the development in Myanmar and instead imposed sanctions on the government of the military regime. The agrarian reform of 1988 forced farmers to grow mainly rice, even if the site conditions were not suitable (Hudson-Rodd & Nyunt, 2001); the following government in 1997 gave privileges to large-scale entrepreneurs, which led to landlessness of poorer people, nonviable small farms, as well as the extension of agricultural land, more mechanical equipment, new seeds, chemical fertilizers, and pesticides (Hudson-Rodd & Nyunt, 2001). The adoption of a new National Land Use Policy in 2015 attempted to recognize customary land rights and the rights of minorities (Bjarnegård, 2020; The Republic of the Union of Myanmar, 2016). The political development and land use policies have also affected the Inle Lake region, where traditional practices such as shifting cultivation can still be found in the upland areas of southern Shan State (Htwe, 2015; Kraas et al., 2017).

#### Population and tourism

With an average population growth of 0.89%, Myanmar is relatively stable (Kraas et al., 2017). Data on population development in the study area are highly variable from one report to another, but a trend of population increase is visible (May, 2007; Michalon et al., 2019; UN Habitat, 2014). According to data from the Immigration and Manpower Department, Taunggyi, Shan State, May (2007), and the recent Census Report from 2014 of the Ministry of Immigration and Population, the population has increased by 30% from 380,000 to about 570,000 within 24 years. The population on the lake itself encompasses about 60,000 inhabitants in 2012. Tourism is also assumed to be a reason for low out-migration (ICIMOD & MONREC, 2017) and more migration towards the area (Michalon, 2014) since this sector provides employment in construction works or as service staff in hotels or restaurants. A rise in touristic foreign visitors to Myanmar came together with the end of political isolation in 2011. The international arrivals went from 600,000 in 2003 to 1 million in 2012 (Ministry of Hotels & Tourism, 2013). This boom in tourism industry especially applies to the Inle Lake, where at least 52 hotels and many new ones under construction can be found in *Nyaung Shwe* and directly on the lake reaching an oversupply (Buijtendijk & Tschunkert, 2016). Nevertheless, it has to be noted that the arrival of international tourists has stopped at the end of the study period caused by the Covid-19 pandemic outbreak in March 2020 and did not resume due to the military coup in February 2021.

#### Rising demand for food and natural resources

The rising demand for food, energy, and water of the growing world population requires increased areas of cropland and higher yields (IPCC, 2019). From 1965 to 2005, world grain harvests have doubled, whereas cropland has only increased by 12% (Foley et al., 2005). The opening of Myanmar to the globalized world also led to increased trade with agricultural goods, which changed its agricultural sector from subsistence to commercially oriented production (Htwe, 2015). Technological progress is contributing to this development. At the Inle Lake, production patterns have especially changed and intensified in the agricultural sector, which is dominated by tomato farming on the floating gardens (Michalon, 2014). Changes in consumption patterns in the area have not been assessed yet in scientific research, but as mentioned above, the demand is assumed to have increased due to population, tourism, and economic growth.

#### Greenhouse gas emissions

Globally, anthropogenic activities have been identified by the IPCC to be the main drivers for the increased atmospheric greenhouse gas (GHG) emissions. Compared to pre-industrial levels, the GHG emissions have increased rapidly, which has led to unprecedented atmospheric concentrations of CO2 in at least 2 million years, and CH4 and N2O in at least 800,000 years (IPCC, 2021). The main source of GHG emissions results from the combustion of fossil fuels, cement production, and flaring, contributing to total emission increase from 1970 to 2010 by 78% (IPCC, 2014). Other main emission sectors are the power industry, buildings, and transport (Crippa et al., 2020). Land use can act as a source and as a sink for CO2 depending on the land management. Land use changes from human activities such as deforestation, livestock, and fertilizer use are estimated to contribute to increased GHG emissions (CO2, CH4, and N2O) by 23% in the period from 2007 to 2016 (IPCC, 2019). Myanmar is among the 100 least emitting countries, who contribute to only 3% of global GHG emissions (Climate Watch, 2016). The main emitting sector is land use change and forestry, whereas agriculture and energy sectors are increasing their share since the 2000s (Climate Watch, 2016).

### Pressures

#### Climate change

The unprecedented GHG emissions from anthropogenic activities have led to observed warming of the atmosphere, ocean, and land (IPCC, 2021). Modeled pathways of global temperature change show that a warming of 1.5 °C and 2 °C will be exceeded during the twenty-first century and result in hot extremes, increased frequency and intensity of floods and droughts, and sea level rise, unless GHG emissions are deeply reduced in the coming decades (IPCC, 2021). In Southeast Asia, human-induced climate change causes a strong variability in precipitation and changed monsoon patterns (IPCC, 2021; Zin & Rutten, 2017), which eventually creates a major challenge of freshwater scarcity with impacts on food production (IPCC, 2014). The country of Myanmar is the second most affected country by climate change from 2000 to 2019 worldwide (Eckstein et al., 2021) with a population, which is vulnerable and exposed to extreme events such as floods and droughts (Rao et al., 2013). In the central dry zone of Myanmar, which is located close to the study area of the Inle Lake catchment, the magnitude of floods and droughts is expected to increase (Rao et al., 2013). In the most recent years of 2010 and 2014, severe droughts were followed by heavy rainfall and floods in 2014 and 2015, leading to landslides, crop failures, and water shortages (Taft & Evers, 2016). The socio-hydrological survey in the study area came to the result that 93% of the participants had noticed weather variability and 68% classify the weather variability as high.

#### Land use change

**Fig. 5:**
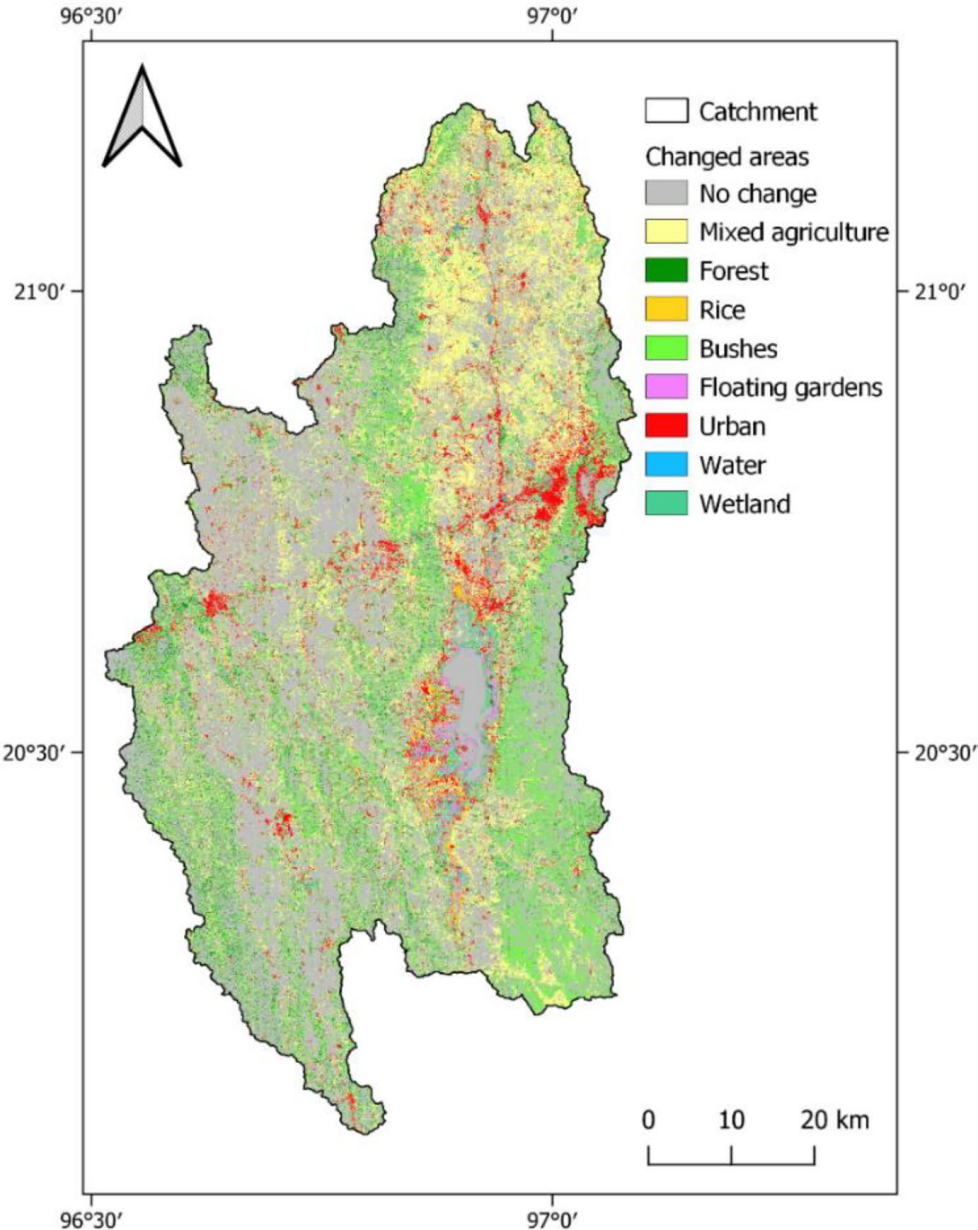
Changed land uses depicted with the land use of 2020 and the areas that were not changed compared to land use of 1990

Political, industrial, and economic development, population increase, and increasing demand for resources are potential drivers for land use change. Several land use change assessments exist for the Inle Lake catchment and a frequently used time span for comparison is 25 to 40 years (Furuichi, 2009; Htwe et al., 2014; Karki et al., 2018; May, 2007; Phyo et al., 2019). Figure 5 shows the changes between the classifications in 1990 and 2020, which are listed in percentage for each class in Table 5.

**Table 5:** Land use classes for 1990 and 2020, share of catchment, and net change

**Land use class**	**Share of catchment in 1990 (%)**	**Share of catchment in 2020 (%)**	**Net change from 1990 to 2020 (%)**
Mixed agriculture	37.29%	50.36%	+ 13.07%
Forest	30.40%	16.95%	− 13.45%
Rice	1.87%	1.71%	− 0.17%
Range-brush	23.78%	19.10%	− 4.68%
Floating gardens/tomatoes	1.29%	1.13%	− 0.17%
Urban	1.11%	6.22%	+ 5.11%
Water	1.54%	0.99%	− 0.55%
Wetland	2.72%	3.54%	+ 0.82%

The most significant changes have occurred in the classes of mixed agriculture and forest. The latter has decreased by about 13%; approximately the same amount by which agricultural land use has increased, which does not mean that all forest was converted to agricultural land. It can be observed that a large part of changed areas has a land use class of range-brush in 2020. This effect might be related to different water availability during the time of the two satellite images (Wilken et al., 2017), but both scenes were taken towards the end of the dry season, so that the phenology should be comparable. Another explanation for this change is the degradation from forest to bushland, for example, from shifting cultivation or other drivers like the demand for wood or climate variability. The conversion of bushland and forest to agriculture is mainly observable in the northern part of the catchment. Shifting cultivation practice seems not to be a main driver for this development in the north, since it is mainly practiced in the western and eastern mountain regions (Htwe et al., 2014). It seems more likely that the general political development, rising demand, and population increase are among the main influences. The two agricultural sub-classes rice and tomatoes have not experienced significant changes, as well as wetland and water. The floating garden’s main growth has already happened before 1990, as confirmed by Htwe et al. (2014). Another trend is urbanization, which is quantified by the classification as a 5% increase.

#### Land use intensification

Land use changes and especially the expansion of agricultural areas reflect the rising demand for agricultural goods and natural resources. Technological progress provides more efficient equipment and possibilities to maximize outputs with the help of irrigation, fertilizers, and pesticides. At the Inle Lake, intensification could be observed in tomato trade, the main crop grown on the floating gardens. According to the land use classification of 2020, the floating gardens cover an area of about 32 km^2^ of the Inle Lake. Tomatoes are grown on 90% of the floating gardens (Michalon, 2014). This farming tradition was already described by Annandale in 1918, whereas it only became an economic sector in the 1960s (Sidle et al., 2007), caused by the improvement of national infrastructure (Michalon, 2014). Another increase was noticed in the 1980s. In the 1990s, it was forbidden to extend further, which led to a densification of the fields (Michalon, 2014). Nowadays, the tomato trade is one of the main activities of inhabitants of the stilt villages on Inle Lake (Personal Interviews, 2020). The floating garden’s hydroponic character makes it possible to detach from seasonal cycles and most farmers indicated that they grow tomatoes in two seasons per year, sometimes even three. This frequent harvest generates high yields, which have in recent years increased with the usage of imported hybrid seeds from China and Thailand (Personal Interviews, 2020). Another trend is the increase of fertilizer input (Personal Interviews, 2020) and the reduced lifespan of the floating gardens. The mean lifespan is between 8 (Michalon, 2014) and 15 (Htwe, 2015) years, but with a high exploitation it can be reduced to about 3 years (Michalon, 2014). The constant addition of seaweed and mud increases the weight and the floating mats sink to the lake ground where they contribute to sedimentation processes (Htwe, 2015). Intensification in other land use classes is mentioned by Htwe et al. (2015) to be especially driven by shortened fallow periods for shifting cultivation, unsustainable and inefficient cultivation practices, and uncontrolled grazing and logging.

#### Extraction of resources

**Fig. 6:**
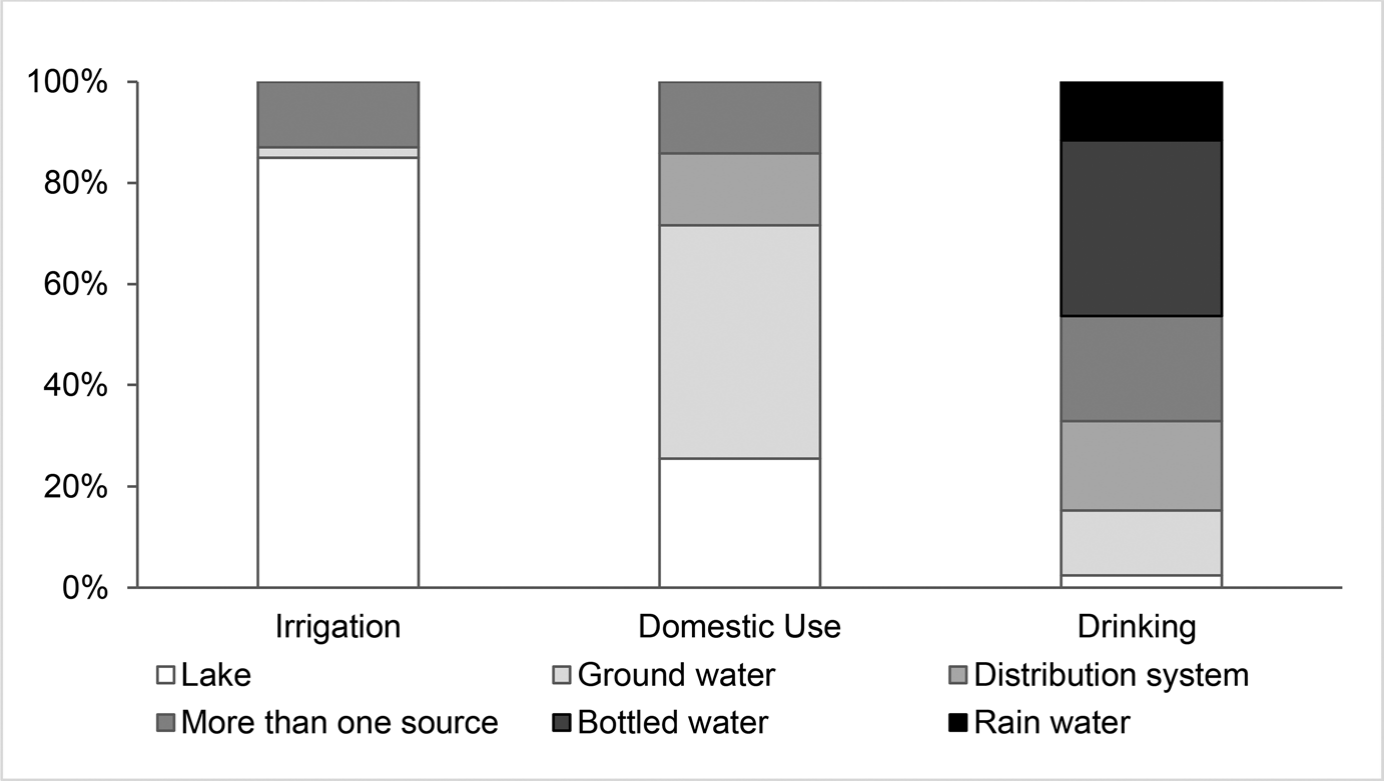
Socio-hydrological survey: Origin of water for irrigation, domestic use, and drinking (Personal Interviews, 2020)

The rising demand and population increase do not only lead to land use change and land use intensification, but also to increased extraction of resources such as wood, water, and fish. Deforestation can be motivated by the demand for agricultural expansion or urbanization. Another pressure on forests is the demand for its resource wood. The socio-hydrological survey showed that among the participants, 82% noticed deforestation within the catchment. The wood is especially used for firing the numerous small sugarcane factories (Htwe, 2015). Wood for domestic consumption is expected to be low since the survey showed that 68% of the households cook with electricity and only 1% still uses fire. Another pressure is the demand for construction wood. The increased construction of hotels and population increase lead to higher demand. Drivers for the increased extraction of water are population increase, rising demand, and industrialization. Figure 6 shows the water resources used for agricultural and household purposes in the lake area indicating that lake water is mainly used for irrigation (Personal Interviews, 2020). Buijtendijk and Tschunkert (2016) mention the hotel industry to be a main pressure on water extraction due to the supply of water to hotel rooms, pools, and garden irrigation. The findings are in agreement with results from a socio-hydrogeological survey by Re et al. (2021) who found that groundwater, which is also distributed in the distribution system, is the main water source for domestic purposes.

A pressure on living resources are the fishing activities on Inle Lake. Nowadays, fishery is mostly restricted to subsistence activities because the population of fish has strongly decreased (Htwe, 2015; Win, 2018; Win et al., 2018), which was confirmed by about 80% of the interview participants. A problem mentioned by the GIZ and Win et al. (2018) is the practice of uncontrolled electro-fishing. In the Inle Lake area it is prohibited in some seasons (Win et al., 2018).

#### Anthropogenic pollution

Potential water polluters are agriculture, industry, households, hotels, and restaurants (Pradhan et al., 2015; Re et al., 2021). Due to the hydroponic agricultural system on Inle Lake, the fertilizers and pesticides applied to floating fields cannot be retained by soil but are transferred directly into the water. The socio-hydrological survey results show that only 3% of floating garden farmers make use of natural fertilizers and 46% indicated to use chemical fertilizers. Use of pesticides is perceived as the main cause of water contamination (Re et al., 2021). Although Myanmar still has a low pesticide use compared to China or Thailand, it is assumed that critical substances are still used in Myanmar due to lack of regulations and an increase in pesticides is projected (Peeters et al., 2015). Industrial activities with pollution risk are cement production, coal power, mining, smithing, and textile production. According to the Pa-Oh Youth Organzation (PYO, 2011), wastewater is released from a coal power plant without monitoring into the *Belu* stream. Additionally, unplanned settlements and the stilt houses on Inle Lake without sanitation infrastructure are main sources of pollution. The socio-hydrological survey results show that about 60% of the participants release their domestic wastewater directly into the lake. Sanitary wastewater is released by 14%, whereas many participants indicated that sanitary wastewater is stored in a septic tank. Spilling of petroleum from the high number of boats on the lake is also reported. Not only the wastewater treatment is inappropriate but also the waste management system, although 86% of the participants have access to it (Personal Interviews, 2020). In *Nyaung Shwe*, 25% of all hotels are served within this waste management system which includes the collection of solid household, restaurant, and hotel waste and burying it or burning at dumpsites (Buijtendijk & Tschunkert, 2016). Another, but presumably smaller, pressure is the practice of Lake Burials. Traditionally, corpses were wrapped and dropped to the lake bottom or buried below the floating gardens. Nowadays, this practice is prohibited, and crematoriums have been built in *Ywama* and *Nam Pan*, but still these are not affordable for some people, who continue the traditional practice (UN Habitat, 2014).

### States

#### Water balance

The pressures of land use change lead to altered states of the environmental, physical, chemical, and biological conditions of which some can be observed in the changed water balance components in Table 6. The same precipitation data was used in both models and the slight net change results from different spatial delineation of the HRUs.

**Table 6:** Water balance components of the SWAT + models based on the land use maps in 1990 and 2020

**Process**	**Unit**	**1990 mode**l	**2020 model**	**Net change**
Precipitation	mm/a	1512	1514	2
Surface runoff	mm/a	225	278	53
Lateral flow	mm/a	145	136	− 9
Water yield	mm/a	370	413	43
Percolation	mm/a	25	20	− 5
Evapotranspiration	mm/a	1113	1076	− 37

**Fig. 7:**
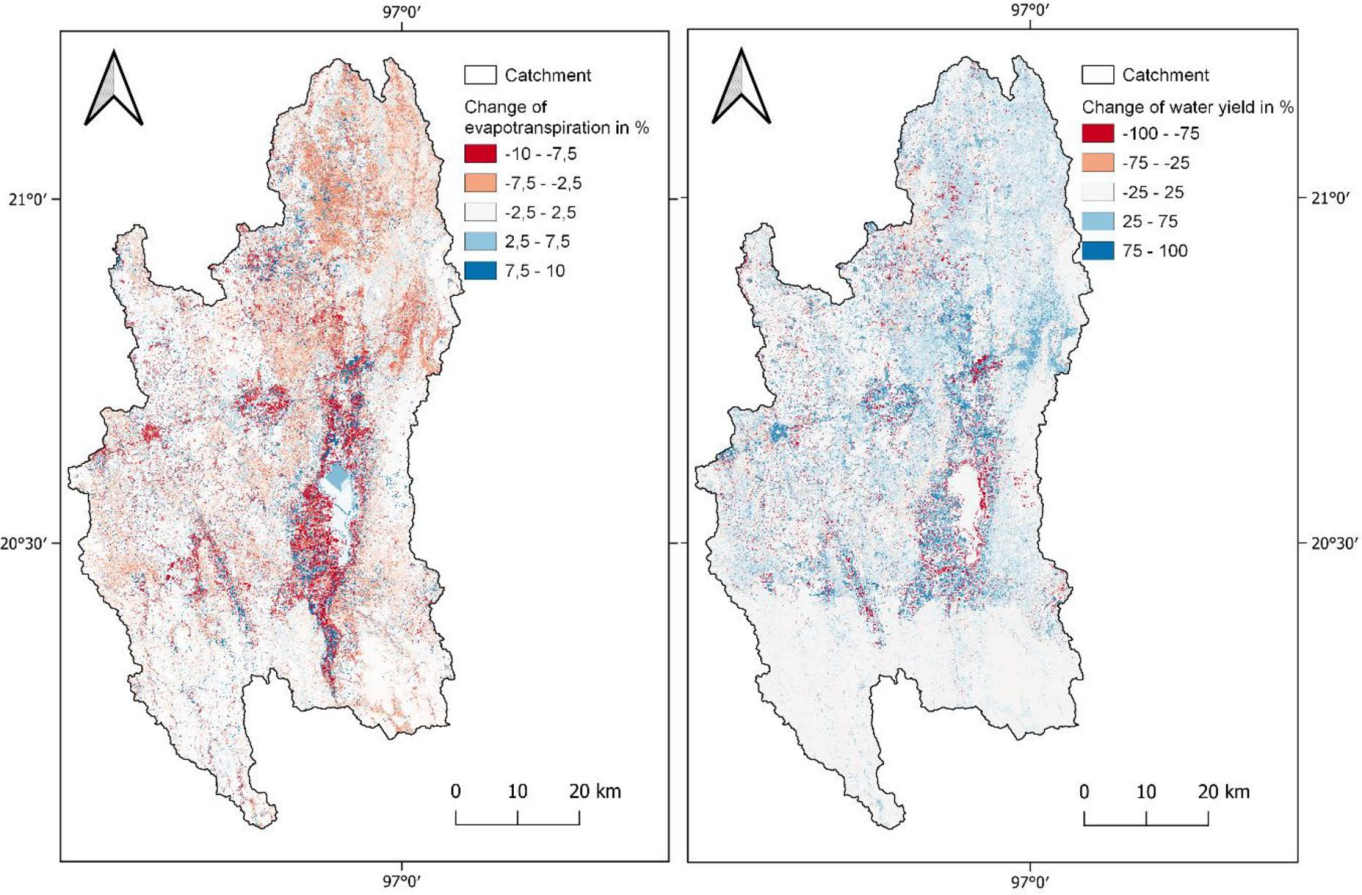
Changes of evapotranspiration and water yield in % per HRU

**Fig. 8:**
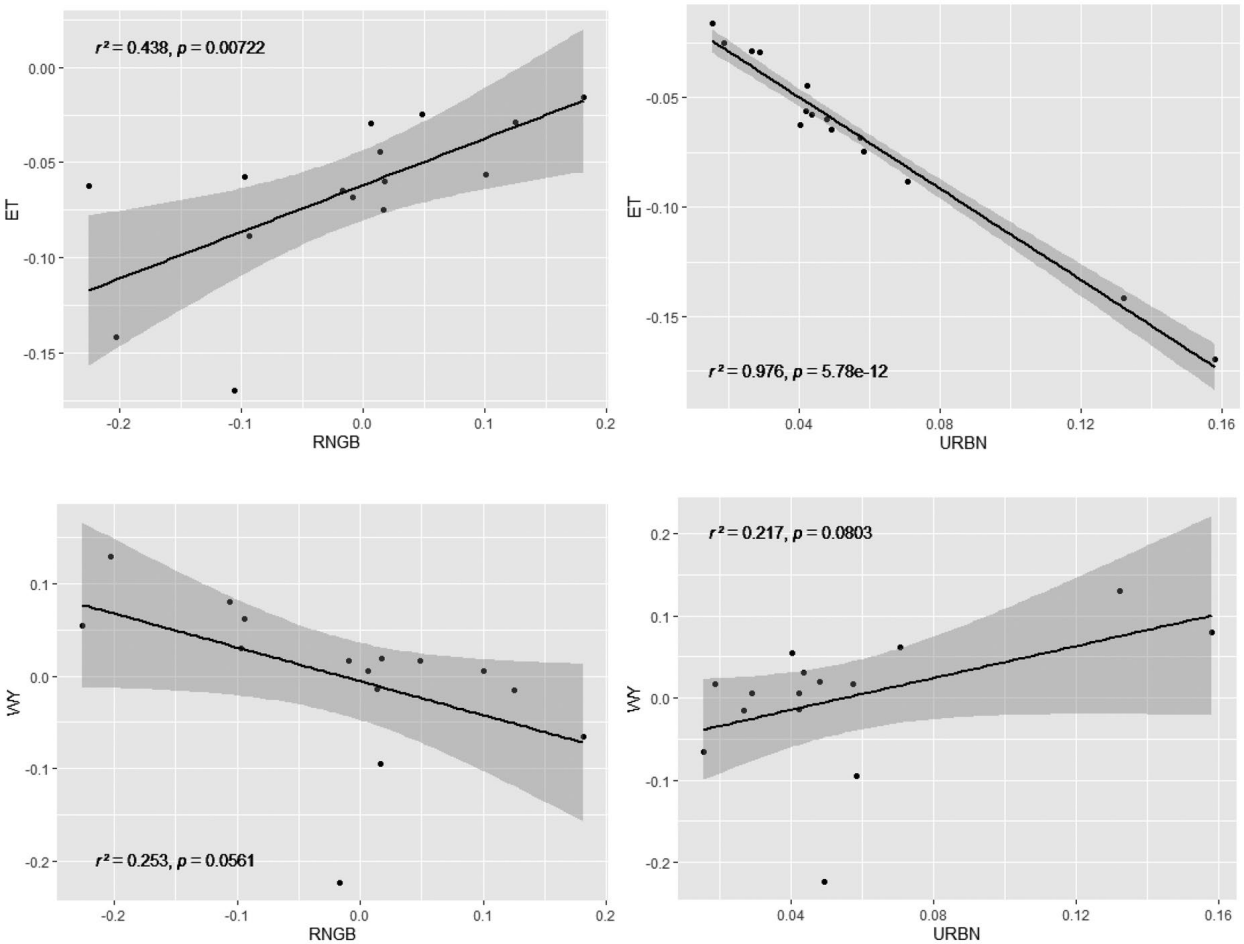
Linear regression of change per subbasin between evapotranspiration, water yield, and urban, range-brush

Overall changes can be seen in the increased water yield and surface runoff by 43 and 53 mm/a. Evapotranspiration has decreased by 37 mm/a. Since the only changed parameter between the models is land use, these developments can be explained by the loss of vegetation and altered management of the soil by increased agricultural activity (Foley et al., 2005). Figure 7 shows the changes per HRU in evapotranspiration and water yield between the two different models. The strongest changes for both ET and WY occur in the north and west. Low changes can be observed in the southern part of the catchment, which is the area that experienced the least land use changes. The relatively high percentage changes close to the lake are due to relatively small initial values. To analyze the influence of land use changes on ET and WY on subbasin level, linear regression analysis was performed, relating the changes in land use to the changes in the water balance components (Fig. 8).

Correlations with *p* < 0.1 between land use classes and the water balance components evapotranspiration and water yield were found for the two land use classes Urban (URBN) and Range-brush (RNGB). Significant correlation (*p* < 0.01) indicates that ET is negatively correlated with URBN (*r*^2^ = 0.98) and positively with RNGB (*r*^2^ = 0.44). A trend of vegetation loss in several subbasins leads to the high losses in ET, which is driven by the population and tourism increase in the area and the construction of new houses and hotels. The opposite applies to water yield in the same areas: an increase of urban land use seems to lead to slightly higher WY (*r*^2^ = 0.22) and an increase of RNGB to slightly less WY (*r*^2^ = 0.25); however, this correlation is not significant (*p* > 0.01). Increasing urbanization and compaction of soil through agricultural activities are potential pressures for increased runoff.

#### Soil erosion

**Fig. 9:**
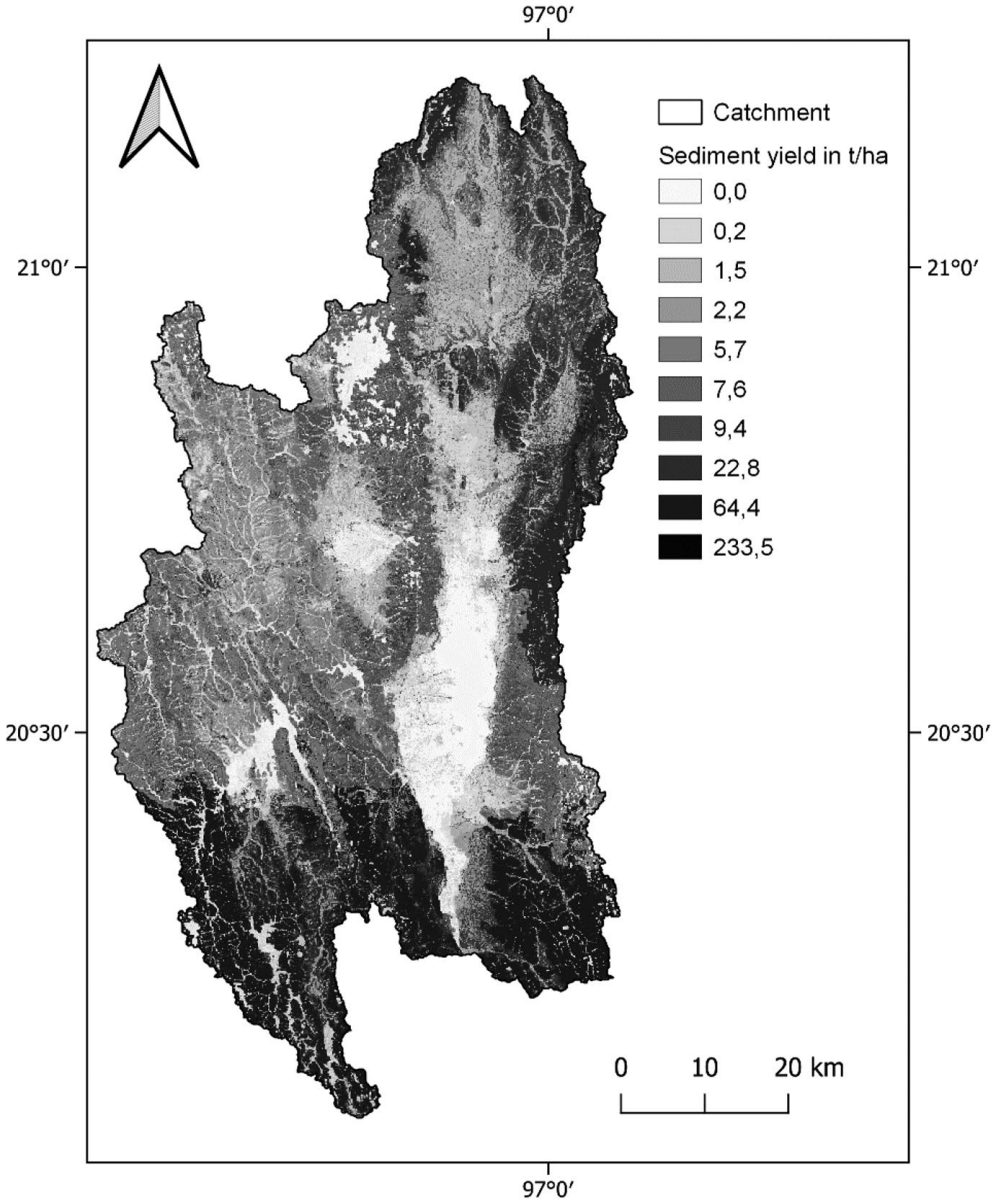
Average annual sediment yield per HRU of the 2020 model (10 quantile classes)

Soil erosion especially affects the central dry zone of Myanmar with an average annual rate of soil erosion from 14.2 t/ha in 2000 to 54.6 t/ha in 2012 (Tun et al., 2015). Located close to the central dry zone, the Inle Lake is threatened by sedimentation, which is caused mainly by the soil erosion in the catchment (Furuichi & Wasson, 2011). The Irrigation Department is responsible for dredging sediments from the major inflowing streams, which have been estimated to amount for 73,440 t/year (Furuichi & Wasson, 2011). The wetlands surrounding the lake are assumed to store more than half of the sediment transported to the lake (Furuichi & Wasson, 2011). Directly on Inle Lake, the following main activities contributing to sedimentation were mentioned in the literature and observed during the fieldwork: the decomposition of abandoned floating gardens, the grazing of water buffalos directly on the shores of the inflowing streams, and the increasing number of motorboat transportation on the lake and within the streams, which cause continuous resuspension of deposited sediment in the shores (Htwe, 2015; Jensen, 2014; May, 2007). Another contributing activity is the removal of macrophytes from the center of the lake for the construction of floating gardens, which causes the lake bottom to be disturbed by detaching of the sediment from the root systems (Sidle et al., 2007). Figure 9 shows the SWAT + 2020 model result for annual average sediment yield (SY) per HRU in the catchment. The mean annual yield is at about 16.7 t/ha in the 2020 model and 15.5 t/ha in 1990. Especially, the north-eastern part of the catchment is affected by erosion risk as well as the southern part. High rainfall erosivity connected to steep slopes applies especially to the southern areas around *Pinlaung*, but not to the northern part of the catchment, which has a more even valley character.

**Fig. 10:**
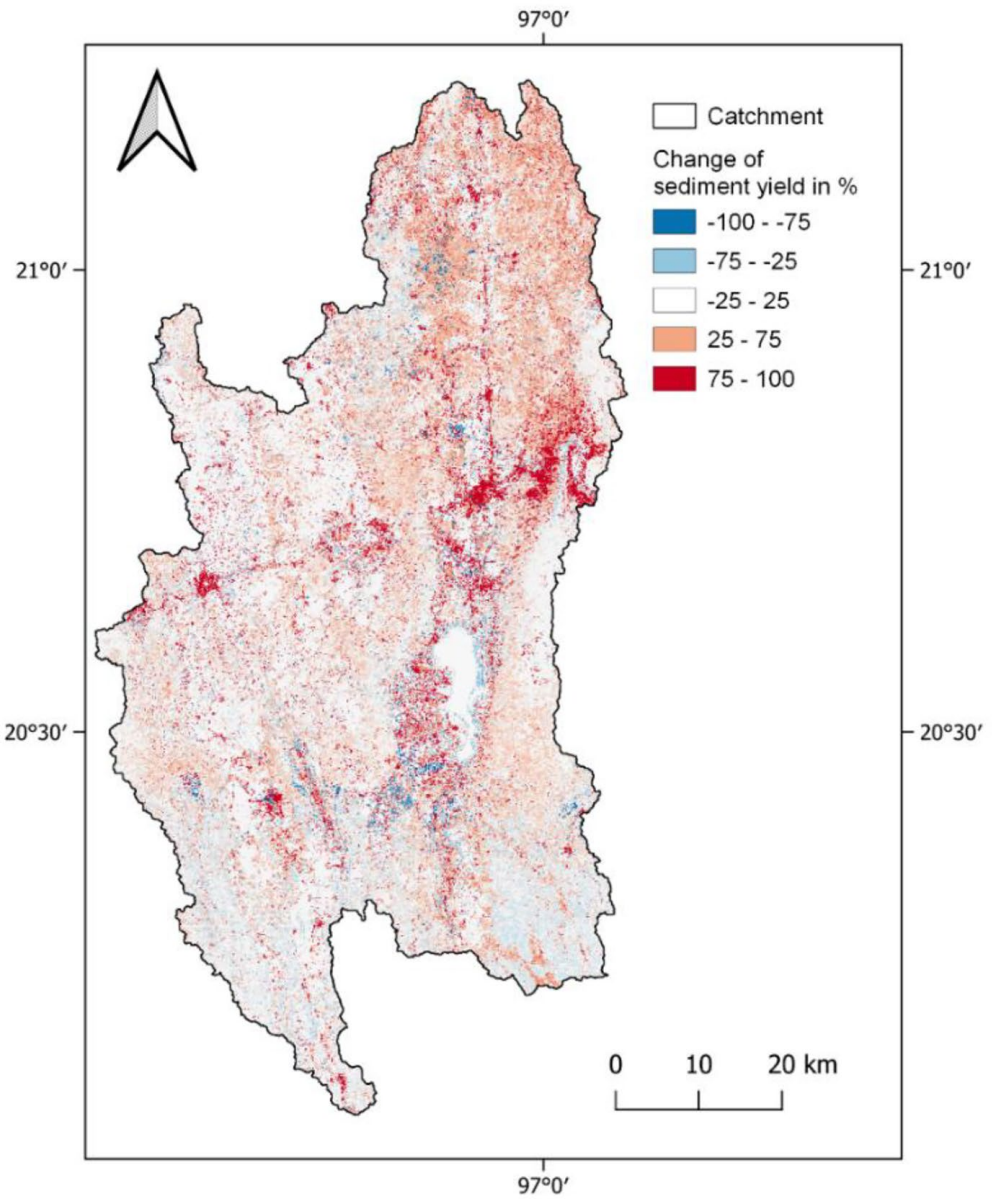
Percentage change of sediment yield per HRU between 1990 and 2020

Figure 10 shows the change in SY between the model result with land use of 1990 and the model result with land use of 2020. The distribution of these changes allows assumptions on the effects of land use change on sediment yield. The highest changes in sediment yield can be observed in the northern and western part of the catchment. As Fig. 9 shows, the absolute values of sediment yield around Inle Lake are close to 0 and the high percent changes (Fig. 10) correspond to relatively small absolute changes. It is remarkable that the area of Upper *Belu* stream in the south has experienced low change, whereas it shows the highest absolute amount of average annual sediment yield. This leads to the assumption that sediment yield in the southern area is mainly naturally caused by the steep slopes and strong precipitation in this mountainous area. Within the northern part of the catchment, pressures on the erosion amount are the urbanization, deforestation, and increased agricultural areas accelerated by land use intensification.

To validate these assumptions, Table 7 shows the mean sediment yield per land use and slope class. The highest values can be found in the classes of forest and range-brush and in slope class 3. This observation leads to the assumption that high slopes, as it is the case in the southern catchment, and high amounts of precipitation lead to the high sediment yield of the SWAT + model in forest and range-brush classes, which are represented by 82% and 73% of their total area on the highest slope class (≥ 8.3%). A regression analysis of SY and the most abundant land use classes in the catchment (AGRL, FRSD, RNGB, and URBN) showed no significant correlation.

**Table 7:** Sediment yield in t/ha per land use and slope class

**Land use/slope class**	**SY [t/ha] SWAT + (2020)**
Mixed agriculture	11.26
Forest	29.18
Rice	2.73
Range-brush	24.18
Floating gardens/tomatoes	1.93
Urban	16.57
Water	0.19
Wetland	9.52
Slope < 2.8%	0.86
Slope ≥ 2.8%	8.20
Slope ≥ 8.3%	29.78

#### Water quality

Potential pollutants from agriculture, industry, households, restaurants, and hotels cause a deterioration of water quality. Within the scope of this work, in situ data was measured at stations 1–8 in the lake and A–D in the four inflowing streams (Fig. 3). The pH values in all stations are slightly alkaline within a range of 7 to 8, which is comparable to findings by Re et al. (2018) and Ballot et al. (2017). Conductivity was between 250 and 550 µS/cm, which was also found by Ballot et al. (2017). The measured values of conductivity and TDS also were in comparable ranges.

In situ data and lab results from December 2019 (Phyo et al., 2022, unpublished report) showed similar results to the measured values from Table 8. In general, the values for TDS and conductivity increased from December 2019 to March 2020 and in each measurement campaign the stations 4 and 5 had the highest rates among the stations on the lake. High levels of turbidity and hardness were measured in several stations. An *E. coli* (*Escherichia coli*) assessment in 2019 close to Kela village showed that the water is contaminated with human feces containing more than 100 MPN/100 ml (Phyo et al., 2022, unpublished report). Contamination with heavy metals is another threat to water quality of Inle Lake. High values were discovered in December 2019 for station 6 with 0.5 mg/l and station 7 with 0.21 mg/l of manganese (Mn) exceeding the limit for drinking standards by the WHO of 0.4 mg/l at station 6. A value of 0.05 mg/l of arsenic (As) in station 8 and 3 also exceeds the limit of 0.01 mg/l, but is still below the national recommended aquatic life criteria by EPA, which constitutes that arsenic contents above 0.15 mg/l indicate chronic pollution and exceeding 0.34 mg/l is acute (EPA, 2020). Heavy metal abundance can also be indicated by total organic carbon (TOC) in C% in sediments (Table 9), which is a parameter for material exchange processes (Seiter et al., 2004). TOC in sediments had a mean value of 12.2% with lowest (6.9%) in station 8 at Inbawkon and highest in station 2 (16.2%) between two hotels. These values are high compared to other findings from authors, for example, in Lake Bosten, China (1.8–4.4% TOC) (Yu et al., 2015) or up to 3.5% in India (Gireeshkumar et al., 2013). As Thin et al. (2020b) found in a mineralogical and geochemical characterization of Inle Lake, the trace elements that are now stored in organic matter could be released in case of anoxic conditions, increasing the threat of transfer into the food chain.

**Table 8:** In situ water quality data

	**Date**	**Time**	**Air T (°C)**	**Water T (°C)**	**pH**	**Conductivity (µS/cm)**	**TDS (mg/l)**
**Station 1**	15.03	09:15	26	24.3	7.74	330	217
**Station 2**	15.03	12:02	37	27.9	7.5	361	239
**Station 3**	15.03	11:40	37	27.4	7.9	276	187
**Station 4**	15.03	12:22	37	27	7.18	429	263
**Station 5**	15.03	11:07	37	25.1	7.01	496	338
**Station 6**	15.03	10:42	35	23.3	7.26	399	244
**Station 7**	15.03	10:12	30	25.9	7	363	245
**Station 8**	15.03	09:35	28	24.6	7.86	350	243
**Station A**	16.03	09:12	24	22.3	7.55	402	268
**Station B**	16.03	10:10	28	24.7	7.28	528	363
**Station C**	16.03	11:17	30	23.5	7.63	406	233
**Station D**	16.03	11:57	34	25.2	7.13	510	324

**Table 9:** C% in sediment from elemental analysis

**Station**	**Type**	**C%**
Station 1	Sediment	12.02
Station 2	Sediment	16.16
Station 3	Sediment	13.17
Station 4	Sediment	11.63
Station 5	Sediment	12.39
Station 6	Sediment	9.18
Station 7	Sediment	15.90
Station 8	Sediment	6.92

Additionally, water hyacinths (*Eichhornia crassipes*) can be used as an indicator for pollution, due to their ability to absorb large quantities of heavy metals from the water (Matindi et al., 2014). The high occurrence of this plant in Inle Lake indicates possible heavy metal availability from anthropogenic sources such as solid waste or wastewater and acts as a source of biomagnification by moving the heavy metals into the food chain.

### Impacts

#### Water resources

Soil erosion shows its impacts on the water resources of Inle Lake with sedimentation leading to an impact of reduction of open water area. This reduction was assessed based on the land use classifications of 1990 and 2020 with the result of 21.33%. The value is comparable to other results such as Sidle et al. (2007) with 32.4% from 1935 to 2000, May (2007) with 27% from 1990 to 2005, or Phyo et al. (2019) with 19.63% from 1995 to 2019. Variations might be caused by impacts of dry and wet years, even though in this case both classifications are based on satellite images from dry seasons. A declining water depth was perceived by the participants of the survey, estimating the water depth in rainy season to about 2.37 m and 0.98 m in dry season (Personal Interviews, 2020). The reduced volume of Inle Lake affects biodiversity and the ecosystems depending on it, as well as the inhabitants of the lake and the downstream areas.

From the water balance in Table 6, it could be seen that percolation decreased by about 4.5 mm annually, so that less water is infiltrated through the soil to recharge aquifers. The groundwater dynamics at Inle Lake assessed by Re et al. (2018) evidence that recharge is sustained by upwelling of deep groundwater circulating in the fractured dolomite bedrock, but the groundwater recharge is currently threatened by exploitation and eutrophication (Re et al., 2018). The availability of water is also affected by climate change, which leads to changes in monsoon patterns and risks of longer drought periods (IPCC, 2021; Rao et al., 2013; Taft & Evers, 2016). On local level, the altered distribution of water can change regional winds and consequently moisture, temperature, and precipitation distribution (IPCC, 2019).

#### Soil, ecosystem, and biodiversity

**Fig. 11:**
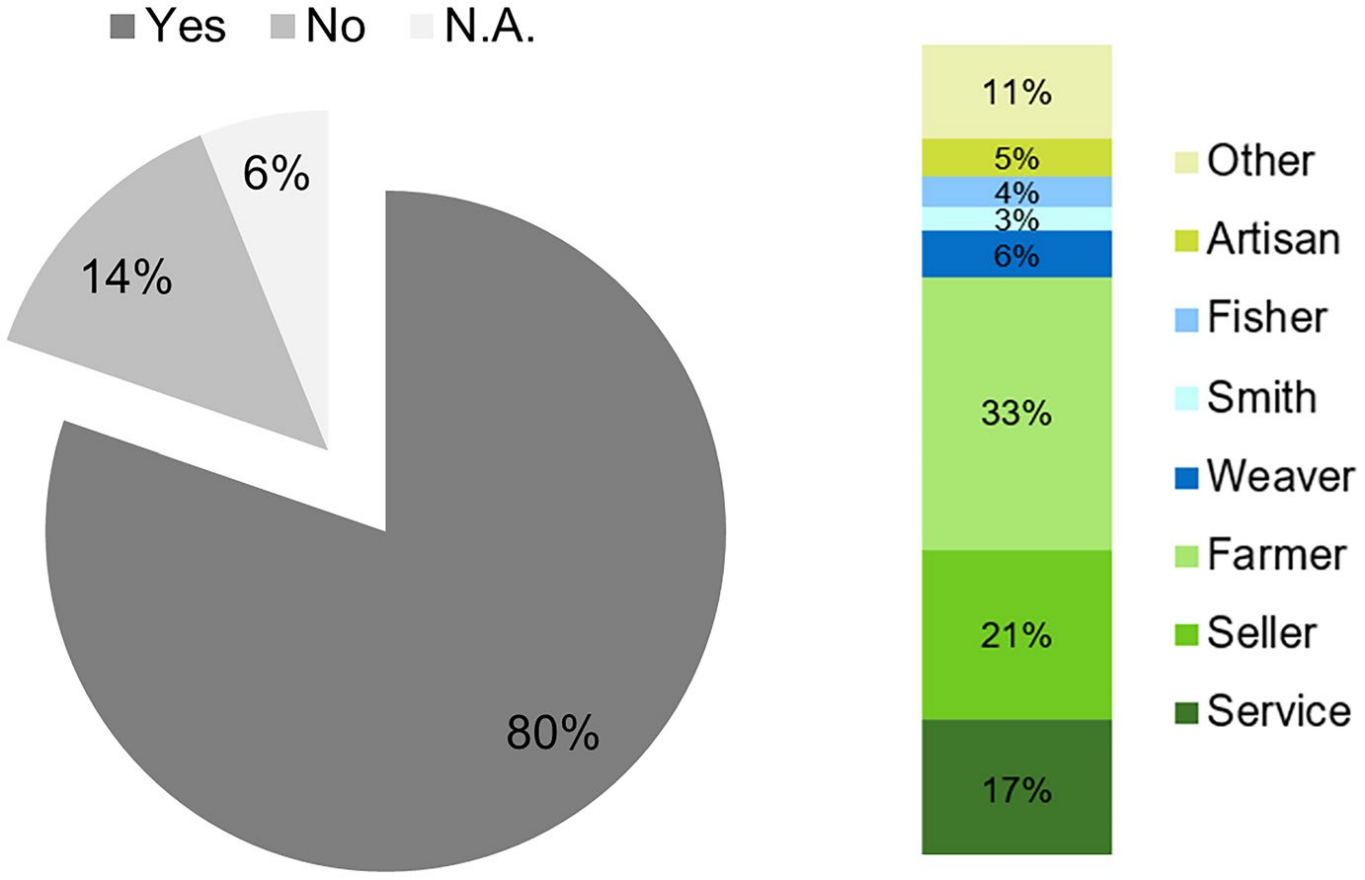
Socio-hydrological survey: Share of people affected by water hyacinths and the profession groups that are affected (Personal Interviews, 2020)

Together with the sediment, erosion also removes nutrients, accelerates acidification, and decreases organic matter content (Htwe et al., 2015). The impact is a decline in regulating ecosystem services, such as degradation of the soil and less fertility (ICIMOD & MONREC, 2017), which leads to reduced yields. The reduced yields again cause land use intensification and increased use of fertilizers and pesticides. This increasing use together with increasing erosion leads to the impact of nutrients being drained into the lake, which eventually causes eutrophication (Sidle et al., 2007). The impacts of climate change are threatening the wetland ecosystem, which is important in the Inle Lake area as a habitat, and to prevent sedimentation and nutrient leaching. According to Karki et al. (2018), the endemic floral and faunal species of Inle Lake are especially threatened by water pollution and decreasing lake water quantity. The declining fish stock in the lake was—according to the interviews—caused by the reduced water quantity (72%). The increasing presence of water hyacinth can be considered as a general threat to local faunal species. Due to its ability to survive under harsh environmental conditions (Matindi et al., 2014), this invasive species threatens not only habitats for local and endemic species, but also affects the economic activities (Fig. 11).

#### Human well-being

The lake inhabitants are not only affected in their mobility and agricultural practices by water hyacinths, but they also experience losses from other reduced ecosystem services functions. Nevertheless, ICIMOD and MONREC (2017) show that the economic situation of the lake inhabitants has improved during the previous years. Security, transportation, public services, health facilities, purchasing power, job market, and education have improved since 2007, whereas the ecosystems services have declined. According to ICIMOD and MONREC (2017), the lake water has not been used for drinking since at least 10 years and about 80% of the participants of the socio-hydrological survey think it is not safe to use the lake water in the household. The decreasing fish stocks and reduced yields in tomato production show eventual economic losses, which could lead to decreased food security and social inequalities. Long-term impacts from the reduction of ecosystem services could affect the tourism sector at Inle Lake. This sector was recently affected by the Covid-19 pandemic, which has eventually stopped international tourism to Myanmar. The transmission of infectious diseases due to global land use change and habitat modification was already predicted by Foley et al. (2005). Increasing tropical deforestation combined with the effects of climate change leads to higher risk of cross-species disease transfer. The pandemic can therefore also be regarded as an impact from the global pressures on the environment and at the Inle Lake it not only affects people’s health directly but also their economic well-being in terms of lost income from the tourism sector.

### Responses

#### Adaptations

Inhabitants of the Inle Lake area are highly aware of the degrading environment and the impacts of this development on their livelihoods. In the case of influences of fertilizers on the water quality, only 2% were not aware of any negative impacts. In most villages, a knowledge-sharing program for environmental protection exists (Personal Interviews, 2020). But responses by the community to cope with negative developments are often adaptations to new conditions. Caused by the pollution of the freshwater of Inle Lake, which was in the past available for drinking, household, and irrigation purposes, the inhabitants were forced to use other sources such as groundwater, rain water harvesting, or bottled water (Personal Interviews, 2020). This adaptation leads back to the cycle by creating a pressure of increased demand for groundwater. Measures against the increasing sedimentation of the lake are taken by the Irrigation Department that dredges the sediment regularly from the main inflowing streams and deposits it at the shores. Additionally, the shores especially along *Belu* stream are secured against bank erosion and small dams were constructed to prevent the transportation of sediments into the lake. These adaptations to the increasing soil erosion do not prevent its cause but might disturb natural processes, such as fish migration, and affect the wetland’s natural function of filtering and retaining water and sediments (Jensen, 2014). Concerning the decreasing fish stocks, the MYSAP program by the GIZ and the Department of Fishery (Win et al., 2018) supports the reintroduction of the endemic *Cyprinus intha* and the practice of aquaculture. The declaration as an ASEAN Heritage Park in 2003 and UNESCO Biosphere Reserve in 2015 were first steps to improve the conservation activities in the 1985 established Inle Lake Wildlife Sanctuary and Bird Preservation Area (Khurtsia, 2015). Conservation activities were implemented under the Inle Lake Conservation and Restoration Plan of MOECAF since 2010. Since cooperation between responsible departments was identified an obstacle, the Inle Lake Development Authority (ILDA) was founded in 2019 to supervise the actual implementation of conservation measures.

#### Recommendations

Coordinated action across all stakeholders from policymakers to local communities is necessary to create sustainable responses. Lack in coordination was found by Uelkes (2015) especially in the non-profit sector (Uelkes, 2015). The Burma Environmental Working Group (BEWG) recommends the adoption of environmental policy and law based on international standards and the development of mandatory laws and regulations in accordance (BEWG, 2011). This requires democracy, transparency in political decisions, and social justice. An UNDP Gender Analysis at Inle Lake showed that projects and sustainable development can only be achieved by empowering women and including them in the decision-making process (UNDP, 2012). The top-down policy also concerns decision-making in land use issues and might prevent local adaptation measures towards changes in climate and environmental conditions. According to a participatory analysis by Castelli et al. (2021), water safety plans, monitoring systems, more information, and infrastructure are needed for a sustainable land and water management. New technologies such as low-cost water quality monitoring systems using a participatory approach (Win et al., 2019) could provide a fast solution for monitoring the water quality and create environmental awareness through citizen participation. Concerning the increased impacts of climate change, solutions need to be found on global level since under current conditions of temperature increase and changing water availability, the limited resources are further under stress. To sustain the water resources, adapted cropping systems or drought-resistant varieties should be implemented to prevent water shortages (Ojima et al., 1994).

## Conclusion

Within the study period of 30 years from 1990 to 2020, the main land use changes in the Inle Lake catchment were the expansion of agricultural land (+ 13%), urbanization (+ 5%), and deforestation (− 13%), which have effects on the water balance by reducing evapotranspiration (− 37 mm) and increasing water yield (+ 43 mm). Linear regression analysis confirmed that the pressure of changed land use is leading to a changed state of the water balance, since the increased urban area has a negative correlation with evapotranspiration on the subbasin level. The application of SWAT + could also show a plausible distribution of sediment yield in the catchment. The overall increase from 15.5 t/ha/a in model with 1990 land use to 16.7 t/h/a in the 2020 model in the whole catchment shows that the main land use changes lead to an increased soil erosion risk. The analysis of change between the two models showed that high average annual sediment yield is in the southern part of the catchment caused by steep slopes and high amounts of precipitation and in the northern and western part accelerated by land use change and environmental degradation.

Not only land use change could be found to have an effect on water resources, but also anthropogenic pollutants such as fertilizers, pesticides, and waste. Sediment assessment shows high values of TOC (7 to 16%), which is an indicator for possible contamination with heavy metals. In situ parameters show higher values in the inflowing streams, which supports the assumption that inputs from agriculture are draining from the catchment into the lake. Nevertheless, the low residence time, the shallow character, and calcite precipitation are reasons for moderate ranges of pollution parameters in the lake.

The results of the quantitative socio-hydrological survey could be used to identify the relevant parts of DPSIR. The main findings from this survey showed that local residents are open for public engagement. There is a high awareness of the local community on the environmental degradation and an urgent need to implement recovery measures.

Through this socio-hydrological approach, the findings of the DPSIR analysis could be confirmed, showing several negative impacts on water resources, soil, ecosystem, and biodiversity, such as the reduction of water area (-21%) and availability of freshwater, which ultimately affect human well-being. The responses to these developments again have effects on drivers and pressures. Therefore, it is necessary to plan recovery measures in a coordinated way with awareness on the processes they affect. Nevertheless, difficulties in implementation and lacking coordination among the stakeholders are encountered and the influence of national politics and global problems such as climate change and the Covid-19 pandemic cannot be mitigated with regional measures.

Our study shows how local, national, and global human activities are intertwined and how they can directly or indirectly influence the availability and quality of water resources of a sensitive freshwater ecosystem such as the Inle Lake. By applying DPSIR together with interdisciplinary methods, we were able to quantify relevant indicators and assess human-water dynamics. This structured approach is a valuable tool to manage and preserve the environment and it was shown that it can be applied in data-scarce regions with reliable results for decision-makers.

## Data Availability

The datasets generated during and/or analyzed during the current study are available from the corresponding author on reasonable request.
